# Supplementary material for: Deletion of wfs1 Impairs Oligodendrocyte Precursor Cells Dorsal Distribution and Myelination Through the wfs1-hmgcs1 Axis in Zebrafish
Source: Biology (Basel). 2026 Aug 16;15(16):1402. doi: 10.3390/biology15161402 (PMC13510151; doi:10.3390/biology15161402)
Supplement: Supplementary file 1 [file biology-15-01402-s001.zip › File S1-Transcriptome sequencing data and differentially expressed genes (DEGs) in wfs1-- zebrafish at 5 dpf/2.KEGG/wfs1KOvsWT/UP/src/dre00562.html]

dre00562


- dre:337236

- Up regulated genes

337236\_2(8.533525538938) 337236(6.56374522648181)

- dre:368717

- Up regulated genes

368717(4.12704824157962)

- dre:678558

- Up regulated genes

678558\_1(6.03678629308862)
- dre:559198

- Up regulated genes

559198(1.99669187801435)

- dre:100006223

- Up regulated genes

100006223(1.86535248914623)

- dre:560753

- Up regulated genes

560753\_1(7.55993178278178)

- dre:571356

- Up regulated genes

571356(1.35534919146634) 571356\_2(3.19114319937523)

- dre:678558

- Up regulated genes

678558\_1(6.03678629308862)
- dre:559198

- Up regulated genes

559198(1.99669187801435)

Close
